# Genetic Architecture of Novel Sources for Reproductive Cold Tolerance in Sorghum

**DOI:** 10.3389/fpls.2021.772177

**Published:** 2021-11-24

**Authors:** Subhadra Chakrabarty, Natalja Kravcov, André Schaffasz, Rod J. Snowdon, Benjamin Wittkop, Steffen Windpassinger

**Affiliations:** Department of Plant Breeding, IFZ Research Centre for Biosystems, Land Use and Nutrition, Justus Liebig University Giessen, Giessen, Germany

**Keywords:** sorghum, GWAS, reproductive cold tolerance, temperate climate adaptation, genetic diversity

## Abstract

Enhancements in reproductive cold tolerance of sorghum are essential to expand growing areas into both high-latitude temperate areas and tropical high-altitude environments. Here we present first insights into the genetic architecture of this trait *via* genome-wide association studies in a broad genetic diversity set (*n* = 330) phenotyped in multi-location field trials including high-altitude tropical (Mexico) and high-latitude temperate (Germany) environments. We observed a high degree of phenotypic variation and identified several novel, temperate-adapted accessions with superior and environmentally stable cold tolerance. Good heritability indicates strong potential for implementation of reproductive cold tolerance in breeding. Although the trait was found to be strongly quantitative, promising genomic regions with multiple-trait associations were found, including hotspots on chromosomes 3 and 10 which contain candidate genes implicated in different developmental and survival processes under abiotic stress conditions.

## Introduction

Sorghum (*Sorghum bicolor* L. Moench, 2*n* = 20) is of vital importance for global food and feed supply. Due to its tolerance to drought and low-input conditions, it represents an essential staple crop and commodity especially in semi-arid regions of Africa, India, Australia, and both Americas. However, as a tropical C_4_ plant, its sensitivity to temperatures below 15°C is a substantial obstruction to successful implementation in both high-latitude temperate climates and tropical high-altitude areas (Singh, 1985). Early juvenile development (e.g., Maulana et al., 2017) and pre-flowering reproductive stage (Brooking, 1976) are considered the most cold-sensitive growth stages. While numerous studies have targeted enhancements of juvenile cold tolerance and analyzed their genetic architecture (e.g., Chopra et al., 2017; Parra-Londono et al., 2018; Schaffasz et al., 2019), comparably little research has focused on reproductive cold tolerance of sorghum to date. Cool temperatures before anthesis are known to induce male sterility in sorghum, leading to complete failure of seed set and grain yield in sensitive genotypes. This phenomenon was first described scientifically by Downes and Marshall (1971). For successful adaption of sorghum to temperate climates as Central Europe, enhancements in reproductive cold tolerance are at least equally important to juvenile cold tolerance (Windpassinger et al., 2015). While farmers can avoid juvenile cold stress by later sowing (albeit at the expense of maturity and yield potential), there is no escape strategy for unpredictable cold spells during reproductive stages.

Brooking (1976, 1979) reported the pre-leptotene and the leptotene as the most cold-sensitive developmental stages, suggesting meiotic problems in microspore mother cells as a possible reason for this phenomenon. Reproductive cold tolerance in a set of 380 sorghum accessions, and identified tolerance sources originating mainly from tropical highlands (e.g., Ethiopia, Uganda), United States and China (Singh, 1985). Further, this study also provided preliminary information on the inheritance of this trait using factorial F_1_ hybrids. Due to the need for cold-tolerant sorghum in the Mexican High Valleys, Mendoza-Onofre (1988) developed cold-tolerant grain sorghum lines with a good local adaption. Over time a steady development of sorghum lines and hybrids with enhanced reproductive cold tolerance has taken place in Mexico (Osuna-Ortega et al., 2000, 2001, 2003; Cisneros-López et al., 2009, 2010; León-Velasco et al., 2009). Furthermore, evaluation of reproductive cold tolerance in the Indian post-rainy season by Krishnamurthy et al. (2014) suggested that *Panicle Harvest Index* (PHI) can be efficiently scored as a proxy for spikelet fertility. In a recent study, Schaffasz et al. (2019) analyzed several traits related to reproductive cold tolerance in a line × tester design, showing a dominant inheritance and heterotic effect in F_1_ hybrids.

However, in spite of the above mentioned progress in breeding cold tolerant varieties and first information about the inheritance of reproductive cold tolerance, there is still no knowledge available about genomic regions involved in its inheritance or the detailed genetic architecture of the trait. This has prevented the implementation of molecular breeding approaches like marker assisted selection, genomic prediction and genome editing so far. In the present study, a broad diversity set (*n* = 330) genotyped using 20K Dartseq markers was utilized for extensive phenotyping of reproductive cold tolerance in multi-environment field trials, aiming at the identification of novel tolerance sources and underlying genomic regions in order to accelerate breeding progress. To our best knowledge, this is the first genome wide association study (GWAS) to date with regard to reproductive cold tolerance in sorghum.

## Materials and Methods

### Germplasm

A *S. bicolor* diversity set consisting of *n* = 330 inbred lines of different origin, type of use (grain, dual-purpose, and forage) and subspecies, comprising all five sorghum races (*bicolor, caudatum, durra, guinea*, and *kafir*) and most of their respective intermediaries, was utilized for the present study. All these lines are photo-insensitive and show a similar, early maturity, allowing for an adequate scoring of seed set traits under cool environments with a short growing season. A representative selection of early-maturing *sorghum conversion lines*, obtained in the 1960s by repeated backcrossing of genetically diverse tropical accessions to a short, photoperiod-insensitive cultivar (Stephens et al., 1967) accounts for 35% (*n* = 117) of the set. A further 22% (*n* = 73) consist of other publicly available accessions from temperate countries, mainly China, Russia, the United States, Turkey, and Hungary. The original germplasm of these accessions and the conversion lines was received from the *United States Department of Agriculture Agricultural Research Service* (USDA-ARS). The remainder of the diversity set consists of 140 diverse breeding lines (grain and dual-purpose sorghum) from a joint breeding program of Justus Liebig University Giessen, Norddeutsche Pflanzenzucht Hans-Georg Lembke KG (Hohenlieth, Germany) and Deutsche Saatveredelung AG (Lippstadt, Germany). The composition of the diversity set is shown in Supplementary Table 1.

### Field Trials

Field trials were conducted at five locations (three in Germany and two in Mexico, Table 1). At Texcoco (TEX17, TEX18), one of the Mexican locations, the diversity set was scored in two subsequent years (2017 and 2018), while at all other locations, the experiments were carried out in 1 year (2017 or 2018). Hence, data from six environments (location × year combination) are available for analyses. The locations of this study represent different mega-environments contrasting in climate, radiation and day length. Among the German locations, Asendorf (AS; located in NW-Germany) and Poel (PL; a small island in the Baltic Sea) have a cool, maritime climate, usually providing harsh conditions for sorghum. In contrast, Gross Gerau (GG) is located in the Upper Rhine Valley and characterized by a warm and sunny climate, being a suitable control environment without cold stress. The locations in Mexico differ strongly from Germany, having shorter days during the growing season but much stronger radiation. While San Juan del Río (SJR; 1,920 m, federal state Querétaro) is considered to be at the altitude limit for commercial sorghum cultivation in Mexico, Texcoco (2,250 m, federal state México) is a tropical high-altitude stress environment for sorghum, providing the lowest minimum temperatures of all locations.

**TABLE 1:** Overview and weather data of the different environments during the duration of the experiments (from sowing until harvest of the panicles).

**Environment**	**Coordinates**	**Altitude**	**Soil type**	**Year**	**Mean temp. (°C)**	**Mean max. temp. (°C)**	**Mean min. temp. (°C)**	**Precipitation (mm)**
PL	53°99’N, 11°47’E	19 m	Loamy sand	2017	16.7	20.2	13.1	301
AS	52°46‘N, 9°01‘E	49 m	Loamy sand	2017	16.1	21.0	11.6	611
GG	49°55‘N, 8°29‘E	90 m	Sand	2018	21.3	28.9	13.7	94 (+150 irrigation)
SJR	20°25‘N, 99°56‘W	1,920 m	Loam	2017	23.0	31.4	14.7	326
TEX17	19°31‘N, 98°51‘W	2,250 m	Loam	2017	16.4	24	8.7	360
TEX18				2018	16.3	23.2	9.4	537

*Environments: Poel (PL), Asendorf (AS), Gross-Gerau (GG), San Juan del Río (SJR), Texcoco 2017 (TEX17), and Texcoco 2018 (TEX18).*

To avoid shading effects by neighbors of different plant height, the inbred lines were split into three subgroups, based on previous scorings of plant height. These subgroups were planted in adjacent but separate blocks at all sites. Within these subgroups, an un-replicated randomized complete block design was used. Entries were grown in microplots, consisting of single rows (2.5 × 0.7 m) at Gross Gerau and double rows (2.5 × 1.4 m) at all other locations, with 0.7 m row spacing and a plant density of approx. 20 plants/m^2^. Plant protection and fertilizer application were executed according to site specific best practice. Per entry, the primary panicles of five healthy plants were covered before anthesis with a transparent Cryovac^®^ bag (330 mm × 750 mm, 15 μm) to avoid cross pollination. These five self-pollinated panicles were considered as biological replications for further analyses. However, due to lodging and insufficient maturity, some plants and entries had to be excluded site-specifically. At maturity, the panicles were harvested with secateurs and dried. The peduncles of each panicle were cut just below the first branches before determining the panicle dry weight. Seed yield (SY) per panicle was measured after threshing. Subsequently, PHI was calculated according to Krishnamurthy et al. (2014) as follows:


PHI= graindryweight(i.e.,seedyieldperpanicle)/panicledryweight(beforethreshing)

Consequently, a PHI value of 0 implies absolutely no seed set, while values close to 1 indicate a high seed set. However, even assuming complete spikelet fertility, PHI will be <1 due to the panicle raw weight. Moreover, seed number (SN) was measured using a Contador seed-counter (Pfeuffer, Germany). In addition to these seed set traits, plant height and start of flowering (in all environments except SJR) were scored on a plot basis.

### Statistical Analyses of Phenotype Data

For statistical analyses of SY, seed number (SN) and PHI, a general linear model was used (IBM SPSS Statistics version 27, IBM Software, Armonk, NY, United States), in which entries (genotypes) and environments (combination of location and year) were considered as fixed effects and replicates (individual plants) as random effects:


$$Y_{\text{ijk}}∼\mu +G_{\text{i}}+E_{\text{j}}+G\,E_{\text{ij}}+R_{\text{kj}}+e$$

where μ represents the population mean, *G*_i_ is the genotypic effect, *E*_j_ is the environmental effect, *GE*_ij_ is the genotype-by-environment interaction, *R*_kj_ is the replicate effect, and *e* is the residual effect.

To compare the levels of genotypic variance obtained in the different environments, ANOVA was also computed separately for each environment, using the following general linear model, where genotypes were considered as fixed and replicates as random effects:


$$Y_{\text{ij}}∼\mu +G_{\text{i}}+R_{\text{j}}+e$$

To determine which environments were significantly different from one another, Student-Newman-Keuls *post hoc*-test was applied.

The heritability was calculated as proposed by Piepho and Möhring (2007) using the following formula:


$$H^{2}=\frac{\sigma_{\text{G}}^{2}}{\sigma_{\text{G}}^{2}+\frac{1}{2}\,\bar{v\,d}}$$

where *h*^2^ represents broad-sense heritability, *σ*^2^*_*G*_* is the genotypic variance calculated by a random effect model considering genotype and environment as random factors, and $\bar{v\,d}$ is the average variance of the difference between two means.

The phenotypic stability of the best performing inbred lines with a complete data set in all stress environments was analyzed using the coefficient of variation (CV) across the mean trait values of a line in each environment.

### Association Mapping and Candidate Gene Identification

The sorghum diversity set was genotyped using DArTseq reduced-representation sequencing^1^ to identify genome-wide single-nucleotide polymorphism (SNP) markers at high resolution. SNPs with more than 20% missing data or a minor allele frequency lower than 5% were removed from the final dataset. After filtering, a total of 21,520 high-quality SNPs remained and were used for downstream analyses.

Analysis of phylogenetic relatedness was conducted with TASSEL version 5.0 (Bradbury et al., 2007) using the neighbor-joining method (Saitou and Nei, 1987). Dendroscope 3.7.3 (Huson et al., 2007) was utilized to visualize the genetic relatedness among accessions in a phylogram. The R package GenABEL (Aulchenko et al., 2007) was used to perform a GWAS for the target traits. To adjust population stratification, a mixed linear model approach was implemented by using the kinship matrix as covariates (Stich et al., 2008). We used a threshold of −log10(*p*) ≥ 3 to minimize type II error and identify SNP-trait association (Gabur et al., 2020).

Candidate genes were identified based on the *S. bicolor* reference genome *v3.1.1* hosted by Phytozome 12^2^, the same reference genome used by Diversity Array Technology to call SNPs. Linkage disequilibrium and haplotype blocks across the entire genome were calculated using the squared allele frequency correlations (r2) between each pair of SNPs, using an LD threshold of *r*^2^ > 0.7 block as described by Gabriel et al. (2002) and implemented using the tool LDBlockShow (Dong et al., 2021).

Genes within haploblocks surrounding trait-associated SNPs with the maximum −log10(*p*) values from GWAS analysis were selected and annotation was checked using the sorghum reference assembly. The gene sequences were further blasted against maize and rice for validation and identification of potential candidates. Additionally, we used the high resolution, open access sorghum QTL Atlas (Mace et al., 2019) to identify and characterize overlapping abiotic stress QTL.

We compared the sequence of *S. bicolor* reference genome *v3.1.*1 and the sequence based on the alternate allele discovered at the SNP position Sb10-5394955 with the recently published sorghum pangenome data (Tao et al., 2021). The gene sequence was selected from the reference genome (*v3.1.*1) and blasted (blastn: 2.2.31; Camacho and Madden, 2013) against the individual thirteen assembled genomes from the sorghum pangenome dataset. The fasta sequence of the results were individually trimmed from the respective genome sequences using bedtools (v2.30.0; Quinlan and Hall, 2010) and aligned using MUSCLE (v3.8.31; Edgar, 2004). The alignment was visualized using SeaView5 (Galtier et al., 1996).

## Results

### Phenotypic Variation for Seed Set Traits

**FIGURE 1:**
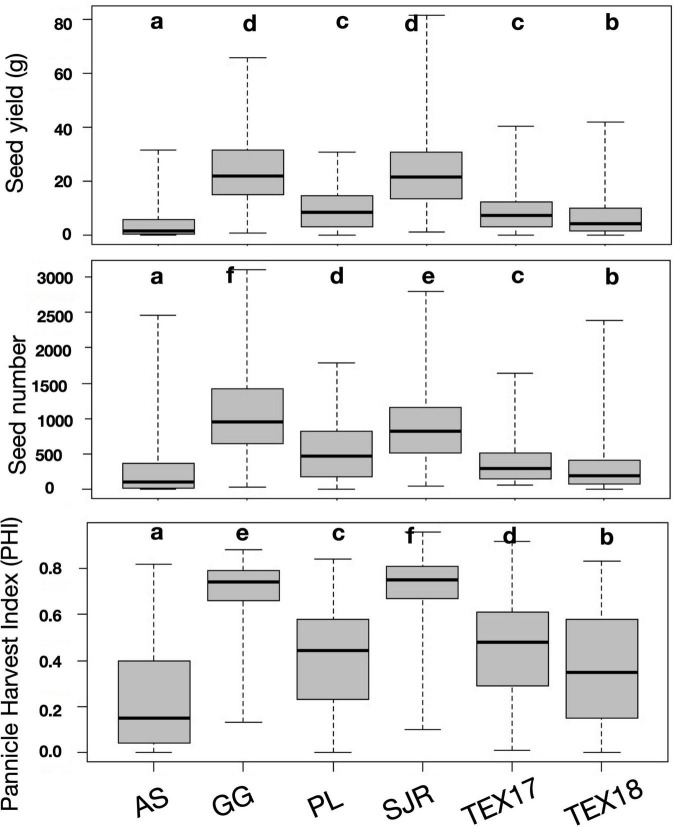
Boxplots showing the variation for the traits seed yield, seed number and panicle harvest index (PHI) across the different environments. Denotation with different letters indicates significant differences as revealed by a Student-Newman-Keuls (SNK) *Post hoc*-Test (α = 0.05).

The temperature conditions and consequently the level of cold stress differed strongly among the phenotyping environments (Table 1), which is also reflected in a high degree of environmental variance on the traits (Table 2). Environments AS, PL, TEX17, and TEX18 showed a significant reduction of PHI, SY, and SN compared to the environments without thermal stress (GG and SJR; Figure 1 and Table 2). In consequence, GG and SJR were regarded as control environments for further analyses, whereas all other environments were considered and pooled as cold stress environments. Highly significant differences among the entries for the scored seed set traits were observed in both environmental groups. However, as expected, the CV was higher for the group “stress environments,” especially for the trait PHI. While genotype × environment interaction between the two control environments GG and SJR was high and of a similar magnitude as genotypic variance, it was lower for the stress environments (depending on the trait, 29–40% of genotypic variance). Heritability estimates for the groups “stress environments” and “all environments” were high for both PHI (*h*^2^ = 0.72 and 0.69, respectively) and SN (*h*^2^ = 0.68 and 0.63, respectively), and somewhat lower for SY (*h*^2^ = 0.60 and 0.51, respectively).

**TABLE 2:** Variances (mean squares) for entries (genotypes), environments (Env), entries × environment (Env) interaction and heritability estimates for the traits seed yield per panicle (SY), seed number (SN), and panicle harvest index (PHI).

**Items**	**All environments**				**Stress environments**				**Control environments**			
	**d.f.**	**SY (g)**	**SN**	**PHI**	**d.f.**	**SY (g)**	**SN**	**PHI**	**d.f.**	**SY (g)**	**SN**	**PHI**
Entries	328	736.18***	1.756 × 10^6^ ***	0.393***	326	434.26***	1.257 × 10^6^***	0.456***	327	755.21***	1.228 × 10^6^ ***	0.076***
CV entries		211.81	229.68	127.89		272.05	286.79	177.77		116.34	115.23	38.83
Env	5	104,344***	143.878 × 10^6^ ***	53.03***	3	7432.54***	19.985 × 10^6^***	9.22***	1	71.88	17.463 × 10^6^ ***	0.359***
Entries × Env	1,520	367.52***	0.654 × 10^6^ ***	0.12***	877	172.99***	0.402 × 10^6^ ***	0.130***	318	809.98***	1.223 × 10^6^ ***	0.064***
Error	6,847	43.2	0.074 × 10^6^	0.014	4,421	25.05	0.054 × 10^6^	0.017	2,426	76.28	0.11 × 10^6^	0.010
Heritability (*H*^2^)		0.51	0.63	0.69		0.60	0.68	0.72		0	0.01	0.17

*Stress environments: Poel (PL), Asendorf (AS), Texcoco 2017 (TEX17), Texcoco 2018 (TEX18); control environments: Gross-Gerau (GG), and San Juan del Río (SJR).*

***P ≤ 0.001.

The race (i.e., panicle architecture) of the genotypes did not influence SY, SN, or PHI (*p* ≥ 0.063), regardless of whether one-way ANOVA was applied for the pooled groups of stress environments, control environments or all environments. Comparing the public material (conversion lines and other public accessions) and the breeding lines developed under Central European conditions as groups, the latter were superior (*p* < 0.001) for all considered traits under the pooled stress environments, while under the control environments a difference between the groups was only detected for SY (*p* = 0.004). The phenotype data of all entries are provided in Supplementary Table 1.

### Trait Correlations

As expected, SY, SN, and PHI were highly correlated with each other (Table 3). However, the correlation of PHI to SY and SN was stronger for the group of stress environments (*r* = 0.880^∗∗∗^ and 0.780^∗∗∗^, respectively) than for the control environments (*r* = 0.647^∗∗∗^ and 0.578^∗∗∗^, respectively). In contrast, TKW was only weakly related to these traits, implying SN as the predominant factor for SY and PHI in all environmental groups. Plant height showed a significant and positive correlation to SY, PHI and – at a lower level especially in control environments- also SN. Whereas TKW correlated with plant height in the control environments, no relation between these traits was found in the stress environments.

**TABLE 3:** Pearson’s correlation coefficient (*r*) among the traits seed yield (SY), seed number (SN), panicle harvest index (PHI), thousand kernel weight (TKW), and plant height (PH) across the different environmental groups [stress environments: Poel (PL), Asendorf (AS), Texcoco 2017 (TEX17), Texcoco 2018 (TEX18); control environments: Gross-Gerau (GG), San Juan del Río (SJR)], based on accession-level mean trait values.

	**All environments**				**Stress environments**				**Control environments**			
**Traits**	**SY**	**SN**	**PHI**	**TKW**	**SY**	**SN**	**PHI**	**TKW**	**SY**	**SN**	**PHI**	**TKW**
SN	0.902***				0.914***				0.880***			
PHI	0.804***	0.723***			0.880***	0.780***			0.647***	0.578***		
TKW	0.169**	-0.157*	0.291***		0.253***	-0.051	0.321***		0.259***	-0.166**	0.259***	
PH	0.380***	0.289***	0.401***	0.122	0.325***	0.306***	0.382***	0.013	0.296***	0.158*	0.269***	0.283***

*P ≤ 0.05, **P ≤ 0.01, and ***P ≤ 0.001.

Trait correlations were inconsistent among the different single environments (Supplementary Table 2). While moderate correlations were observed between AS, PL, TEX18 and SJR, TEX17 showed only weak correlations to the other environments. Surprisingly, GG showed no correlation to any of the other environments (*p* ≥ 0.103). Pearson’s correlation coefficients of *r* = 0.343^∗∗∗^ for SY, *r* = 0.442^∗∗∗^ for SN and *r* = 0.385^∗∗∗^ for PHI were observed between the pooled groups of stress and control environments.

The effect of flowering time on the yield-related traits was generally weak and inconsistent over the environments (Supplementary Table 3), as expected due to the vast ecogeographical variation between the testing locations. For the control environment GG, there was no effect of flowering time at all (*p* ≥ 0.053). Whereas a significant and moderately negative correlation between days to flowering and TKW was observed for the temperate, short-season stress environments of AS and PL, this effect was much weaker for the tropical stress environments (insignificant for TEX17 and weak for TEX18). Also for PHI, later flowering genotypes showed a slight disadvantage at three out of four stress environments. In contrast, later flowering resulted in no penalty for SN nor SY (with the exception of location PL).

A substantial proportion (*n* = 110) of the diversity set had previously been extensively scored for juvenile cold tolerance and early vigor, in independent field trials and climate chamber experiments (Schaffasz et al., 2019). However, only few, weak and inconsistent correlations between juvenile and reproductive cold tolerance could be found. Seedling emergence of an early-sown field experiment in Giessen, Germany in 2014 showed a positive, but weak correlation to mean SN (*r* = 0.197^∗^) and PHI (*r* = 0.200^∗^) of the pooled stress environments in the present study. Similarly, seedling emergence under controlled cold stress (13/10°C) was also positively related (*r* = 0.204^∗^) to mean PHI of stress environments. In contrast, mean juvenile biomass of four field trials (Schaffasz et al., 2019) correlated negatively with mean SN (*r* = −0.221^∗^) and mean PHI (*r* = −0.198^∗^) of the pooled stress environments in the present study. Furthermore, leaf greenness, a score for photosynthetic activity under controlled and prolonged cold stress, was negatively related to PHI as well (*r* = −0.238^∗^).

### Accessions With Superior Reproductive Cold Tolerance

**FIGURE 2:**
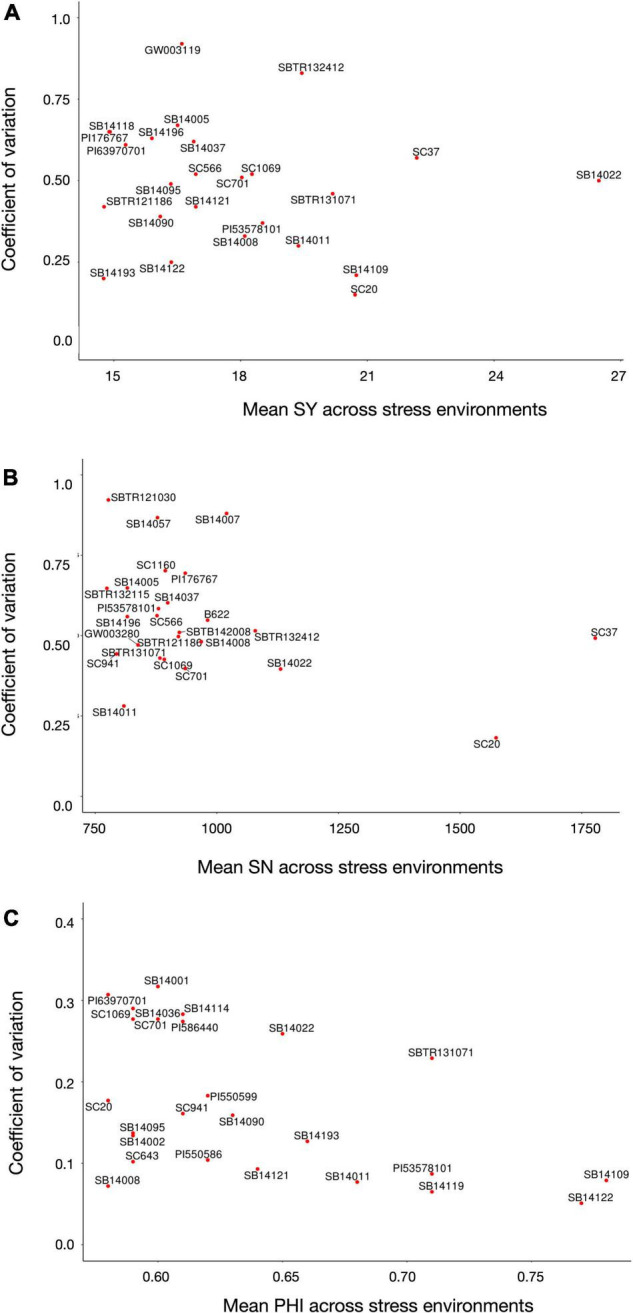
Mean values and stability, depicted as coefficient of variation across the mean trait values of a line in each environment (low values indicate a higher stability), of the top 5% (*n* = 12) best performing genotypes with complete dataset over all four stress environments for **(A)** seed yield per panicle, **(B)** seed number, and **(C)** and PHI.

To identify accessions with a superior and stable reproductive cold tolerance, the 5% (*n* = 12) best performing genotypes for each trait, based on their mean over all four stress environments [for which only entries with a complete dataset (*n* = 240) were considered], were further dissected for their stability, expressed by the CV across the mean trait values of a line in each environment. Figure 2 shows that breeding lines SB14109 and SB14122 attained the highest PHI scores. Among the public material, accessions PI53578101 (from United States), PI550586 and PI550599 (both from Russia) showed the best results. Regarding SN, the conversion line SC37 (originating from Ethiopia) reached the highest mean value, but a rather low environmental stability due to a poor performance in Texcoco2017. In contrast, SC20 (also originating from Ethiopia) showed a very high environmental stability at the expense of just a slight reduction in mean performance, hence appearing to be the preferable tolerance source. Further, public lines SC701 (from Sudan) and PI176767 (from Turkey) ranked among the top 5% with regard to reproductive cold tolerance. As expected due to the high trait correlations, SC37, SC701, and SC20 (again at a superior environmental stability) were among the top-performers for SY. Breeding line SB14022 reached the highest mean SY, but showed a rather high environmental fluctuation. Moreover, public lines SC1069 and SC566 (both from Nigeria) were among the top performers for SY under cold stress. Chinese *kaoliang* types (*n* = 15), which are known for their superior juvenile cold tolerance, showed only moderate levels of reproductive cold tolerance (Supplementary Table 1). This finding is concordant with the low and inconsistent correlation between juvenile and reproductive cold tolerance (see section “Trait Correlations”).

As presented in Table 4, ten lines ranked among the top 5% of performers for more than one trait. Of these, line SB14022 excelled in all three traits simultaneously and is a strong candidate for further focus in breeding.

**TABLE 4:** Overview of the lines ranking among the top 5% performers for more than one trait related to reproductive cold tolerance.

**Genotype**	**Description**	**Origin**	**Among top 5% for traits**
PI53578101	Public accession	United States	PHI, SY
SC20	Conversion line	Ethiopia	SN, SY
SC37	Conversion line	Ethiopia	SN, SY
SC701	Conversion line	Sudan	SN, SY
SB14008	Breeding line	Germany	SN, SY
SB14011	Breeding line	Germany	PHI, SY
SB14022	Breeding line	Germany	PHI, SN, SY
SB14109	Breeding line	Germany	PHI, SY
SBTR131071	Breeding line	Netherlands	PHI, SY
SBTR132412	Breeding line	Netherlands	SN, SY

### Phylogenetic Diversity of Cold Tolerant Lines

**FIGURE 3:**
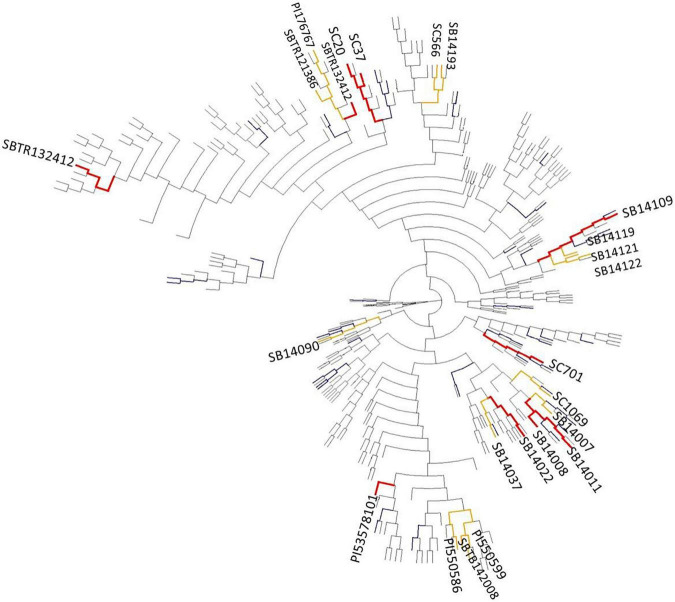
Phylogram constructed using the neighbor-joining method, showing the genetic relatedness in the diversity set (*n* = 330) and among lines with superior reproductive cold tolerance (colored bars). Genotypes ranking among the top 5% of performers for more than one cold tolerance trait (Table 4) are highlighted in red, while genotypes that are among the top 5% for a single trait (Figure 2) are highlighted in orange. Genotypes with a mean panicle harvest index (PHI) in the upper 5–25% quantile in the pooled stress environments are colored in blue, unless they were previously assigned to one of the two previous groups.

The phylogenetic relatedness of the diversity panel, and the localization of genotypes with superior reproductive cold tolerance is depicted as a phylogram in Figure 3, and as a principal component analysis in Supplementary Figure 1. Genotypes with superior reproductive cold tolerance tend to cluster within related branches. However, these branches are rather evenly distributed across the phylogram, indicating that sources of reproductive cold tolerance are not confined to a particular origin.

### Association Mapping and Candidate Gene Identification

**FIGURE 4:**
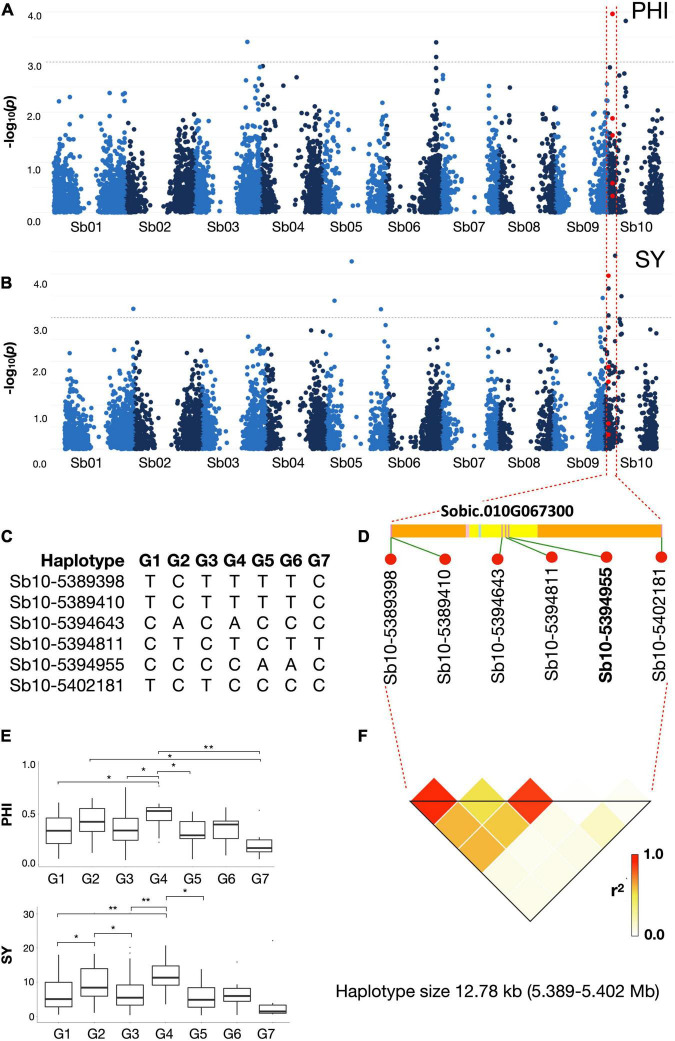
Association mapping of **(A)** panicle harvest index (PHI) and **(B)** seed yield (SY) on chromosome Sb10. Red dots represent a haplotype region spanning just 12.78 kb (5,389,398–5,402,181 bp) including SNP marker Sb10-5394955 which is significantly associated with both PHI and SY under all combined cold stress environments. The gray dotted horizontal line indicates a threshold of genome- wide cut-off at -log10(*p*) > 3.0. The SNP markers with the highest *p* values for each trait were used to define a haplotype region with linkage disequilibrium (LD) to the traits. **(C)** shows the seven haplotype groups (G1 – G7) present in the region. **(D)** The SNP Sb10-5394955 (marked in bold) lies in the coding region of gene Sobic.010G067300 (yellow: CDS; light blue: Intron; pink: UTR; orange: Intergenic region) and **(F)** shows the haploblock around this region. **(E)** *Post Hoc* result for the seven haplotype groups for traits PHI and SY. Boxplots indicate presence of significant difference between haplogroups G1-G4, G4-G3 and G5-G4 for both PHI and SY under all cold stress environments combined.

Using pooled phenotype data from the stress environments AS, PL, TEX17 and TEX18, GWAS identified a total of 55 marker-trait associations for PHI (5 associations), SN (21), SY (9), TKW (9), DTF (1), and PH (10), respectively, (Figure 4 and Supplementary Figures 2, 3). Full details of chromosome positions of identified MTAs (marker trait associations) for each trait, including explained variance (*R*^2^) values and *p*-values, are provided in Supplementary Table 4. Due to the high phenotypic correlations among the SY traits, some overlapping associations were detected between the traits under different environments (Supplementary Table 5). The average phenotypic variance explained (*R*^2^) was 4.34 for the stress environments.

Considering the most relevant traits in regard to reproductive cold tolerance, namely PHI and SY, marker-trait associations were identified on chromosomes Sb01, Sb05, Sb06, Sb09, and Sb10 (Supplementary Figure 2 and Supplementary Table 4). SNP marker Sb10-5394955 on chromosome Sb10 was associated to both PHI and SY (and also associated with TKW) in the cold-stress environments (Figure 4).

The SNP marker Sb10-5394955 on chromosome Sb10, which showed multiple cold-related trait associations to PHI, SY, and TKW, showed relatively weak LD to the adjacent SNPs on either side (*R*^2^ < 0.8; Figure 4), indicating recombination within the haploblock region. Based on the *S. bicolor* reference genome assembly (*S. bicolor* reference genome *v3.1.1*; https://phytozome.jgi.doe.gov/pz/portal.html#!info?alias=Org_Sbicolor), the haplotype block containing Sb10-5394955 overlapped with the gene Sobic.010G067300 and no other genes were present in the region. Strong synteny to the very well-annotated public reference genomes of rice^3^ and maize (B73 RefGen_v5; https://www.maizegdb.org/genome/assembly/Zm-B73-REFERENCE-NAM-5.0) was exploited to obtain putative functional information for this gene. Protein sequence BLAST of the genes present in syntenic rice and maize chromosome regions revealed that *Sobic.010G067300* is a leucine-rich repeat receptor-like protein kinase gene. It has been found to be critically involved in cold response in rice and soybean (Gao and Xue, 2012; Yang et al., 2014).

Haploblocks surrounding each significant SNP were further investigated using the sorghum QTL Atlas (Mace et al., 2019) for overlaps to known QTL for relevant traits in other sorghum populations. QTL overlapping with the associated markers were identified for all traits under both stress and stress+control conditions (Supplementary Table 6). On each of chromosomes Sb03 and Sb10 we identified four overlapping, previously identified QTL related to cold stress overlapping the haploblock regions of traits PHI and SY (Supplementary Figure 4).

## Discussion

### Phenotypic Variation for Reproductive Cold Tolerance in Sorghum

The high amount of phenotypic variation for reproductive cold tolerance observed in the present study, along with satisfying heritability estimates and identified tolerance sources with high environmental stability, suggests that a robust breeding progress for this crucial adaptation trait is feasible, even though marker-assisted selection will be challenging due to its quantitative inheritance. The cold stress environments chosen for this study represent contrasting mega-environments (temperate vs. tropical). While the lowest minimum temperatures occurred at the Mexican highland station Texcoco, the most severe stress reaction of the plant material was observed in the maritime, high-latitude environment of Asendorf (Germany). As already outlined by Schaffasz et al. (2019), this shows that other factors besides minimum temperatures also play a role. For locations Asendorf and Poel, lack of radiation, high moisture and suboptimal daily temperatures of frequently <20°C implied additional stressors, while at Texcoco radiation was not limiting and the daily temperatures were also more favorable. Nevertheless, despite these climatic differences, satisfactory heritability estimates under stress were observed for all traits (*h*^2^ = 0.72 for PHI, 0.68 for SN and 0.60 for SY). Indeed, Schaffasz et al. (2019) reported even higher heritability estimates (*h*^2^ > 0.80 for PHI, SN, SY) for F_1_-hybrids across more or less the same set of environments as those used in the present work.

Panicle harvest index showed the highest heritability and proved to be the most suitable and reliable trait for scoring reproductive cold tolerance in sorghum, representing an efficient proxy for spikelet fertility by reducing the effects of different panicle sizes (i.e., spikelet numbers) considering panicle raw weight. In contrast to our expectations, the results showed that PHI was not influenced by race, which in sorghum is expressed in terms of contrasting panicle architecture. However, the use of PHI as a proxy for spikelet fertility might at least theoretically be distorted to a degree by compensating effects of increased TKW in panicles with poor seed set and, hence, less sink competition. The lack of negative correlation between TKW and SN for the stress environments seems to support this hypothesis, in contrast to the control environments, where a weak negative correlation between these traits was observed as expected (Supplementary Table 3). However, comparing the correlation between PHI and TKW (*r* = 0.321^∗∗∗^) vs. PHI and SN (*r* = 0.780^∗∗∗^) reveals that the influence of SN on PHI is much higher. In a study on sorghum heat stress, which can reduce seed set in the same way as cold stress, Singh et al. (2015) also found that the effect of stress on seed-set percentage was much higher than on TKW, excluding relevant compensation effects of an increased seed mass. Furthermore, there are reports suggesting a determination of maximum seed size of sorghum prior to anthesis based on meristem size (Yang et al., 2009).

In consequence, SN itself represents also an efficient score for reproductive cold tolerance. While it is not affected by possible fluctuations of TKW, it is obviously strongly determined by spikelet number, so that for actual spikelet fertility, it seems a less accurate score than PHI. Though, from a breeder’s point of view, a high spikelet number is desirable, reflecting satisfying yield potential, so that SN might be preferred over PHI for selection. Altogether, both PHI and SN showed a very high correlation to SY (*r* = 0.880^∗∗∗^ and 0.914^∗∗∗^, respectively) in stress environments. Since SY itself had a somewhat lower heritability, PHI and SN may represent preferable surrogate traits for selection.

Differences in flowering time can potentially complicate comparisons of reproductive cold tolerance levels across divergent genotypes, since they can lead to possible escape of earlier or later flowering genotypes from brief cold-weather conditions at the most critical growth stages. In this regard, tropical highland locations like Texcoco are particularly suitable selection environments due to their consistently low and generally stable minimum temperatures during reproductive phases, whereas temperatures tend to be more fluctuating in temperate areas. However, also for Asendorf and Poel, only weak effects of flowering time were observed on stress symptoms. We observed that later flowering entries tended to have a lower TKW in these environments, due to their shorter grain filling period. This also implies a minor disadvantage in terms of PHI, whereas SN was not influenced by flowering time.

The observed positive correlations between plant height and SY traits are in line with earlier reports (e.g., Jordan et al., 2003; George-Jaeggli et al., 2011). Except for TKW, which was probably distorted by the aforementioned compensation effects for reduced spikelet fertility under stress, the correlations with plant height were somewhat higher for the group of stress environments. The increased availability of stem reserves in taller sorghum, which is especially beneficial under stress (Blum et al., 1997), is believed to be the main reason for this phenomenon.

### Tolerance Sources

We identified several inbred lines with superior and environmentally stable reproductive cold tolerance. The diversity panel consisted of both publicly available material (sorghum conversion lines and other public accessions) and breeding lines developed under temperate conditions of Central Europe. As expected due to their breeding history with selection in temperate environments, the latter group performed significantly better under stress conditions. However, some public accessions also ranked among the top 5% for the stress-related traits we evaluated. These superior public accessions were of diverse origin, spanning accessions from temperate (Russia, Turkey, United States) to tropical (Ethiopia, Nigeria, Sudan) countries. While the Ethiopian highland is a known diversity center for cold tolerance (Singh, 1985), the identification of high reproductive cold tolerance in Nigerian sorghum materials may seem surprising.

However, the fact that tolerance sources from both groups (public accessions and private breeding lines) were found among genetically diverse branches over the entire phylogram (Figure 3) suggests a polyphyletic origin of reproductive cold tolerance.

### Detection of Candidate Genes From Association Mapping and Overlapping QTL

The results of our study indicate that reproductive cold tolerance in sorghum is a quantitative and complex trait. This finding is consistent with the description of reproductive cold tolerance in sorghum as a heterotic trait (Schaffasz et al., 2019) and also in line with studies on the reproductive cold tolerance of rice (Jiang et al., 2010; Mori et al., 2011). Since rice diverged from maize and sorghum 50–70 Mya, it has been used as a model for sorghum using a comparative genomic approach (Roulin et al., 2009). The candidate genes discussed hereinafter have been well studied in rice, maize and other crops, indicating that a similar function in sorghum can be expected.

To detect regions associated with reproductive cold tolerance traits, we applied a combination of GWAS and comparative combined-QTL analysis, utilizing a published Sorghum QTL Atlas (Mace et al., 2019) as a reference. Combined-QTL analysis including results from different environments and populations yields a better overview of the genetic control of a trait than any single study (Rong et al., 2007). Since our study comprised environments with and without cold stress, separate analyses were performed to compare QTL detected only in cold stress environments with those detected in all (stress and control) environments. Considering cold stress environments alone, the QTL Atlas (Mace et al., 2019) revealed QTL related to abiotic stress tolerance (Supplementary Table 7 and Supplementary Figure 4) for traits such as emergence rate, seedling vigor and survival that overlapped with our identified associations. However, when focussing on only the associations consistent between the traits PHI and SY, identified in the present study as the two trait complexes most impacted by reproductive cold tolerance, we found overlaps with known QTL for dry matter growth rate, chlorophyll content and chlorophyll fluorescence. Obviously the relatively low resolution of the QTL Atlas means these findings are preliminary, however, they provide a starting point for future functional studies to dissect the genetic and physiological basis of the identified QTL.

As expected due to the high phenotypic trait correlations, GWAS revealed multiple overlapping loci among the considered SY traits. This strengthens our confidence of associations not being false positives (Shen and Carlborg, 2013; Boyles et al., 2016). While some common associations between SY traits and DTF or plant height were also observed, in line with the significant phenotypic correlations among these traits, the majority of associations for reproductive cold tolerance were not linked to a taller stature or earlier flowering. Hence, the breeding of short-statured grain sorghum with enhanced cold tolerance and maturity adapted to the temperate target environment appears to be feasible using the stress tolerance sources we identified. Presence of SNP marker highly associated to cold tolerance within the gene *Sobic.010G067300*, a leucine-rich repeat receptor-like protein kinase further enhances our hypothesis of this genomic region being involved in reproductive cold tolerance. A novel allele of the gene was found to be enriched in breeding lines selected for adaption to temperate climates of central Europe. Based on the results of this study, it can be speculated that the haploblock region around Sb10-5394955 may be involved in temperature adaptation of *S. bicolor* and could be of interest for future breeding efforts.

## Conclusion

Reproductive cold tolerance is a crucial trait to expand sorghum production into both high-latitude temperate areas and tropical high-altitude environments. The present study is the first to analyze this trait in a broad diversity set using data from multi-location field trials including tropical high-altitude and temperate environments. Satisfying heritability estimates and novel, temperate-adapted tolerance sources of polyphyletic origin suggest that robust breeding progress to improve reproductive cold tolerance is feasible.

More detailed genetic dissection of reproductive cold tolerance related traits can help to understand the physiological control of the trait and contribute to more targeted selection. In this study, several significant marker-trait associations were identified. Combining local LD analysis with QTL data from previous studies and synteny to potential candidate genes in rice and maize helped narrow down two interesting marker-trait associations to specific genomic regions involved in cold stress response. One of the promising genomic regions include hotspot on Sb10 which contain a functionally-relevant candidate gene involved in developmental and survival processes under abiotic stress conditions. Future studies to dissect the impact of allelic variants of this gene under contrasting environments and conditions can provide more precise information, functional markers and potential gene editing targets for applied breeding.

## Data Availability Statement

The raw data generated during and/or analyzed during current study have been deposited in NCBI database (https://www.ncbi.nlm.nih.gov/) under the project PRJNA775860.

## Author Contributions

SC conducted data analysis, interpreted the results, and wrote the manuscript. NK conducted data curation and contributed to data analysis. AS planned and oversaw field trials and data collection. RS received the funding and edited the manuscript. BW received the funding and contributed to devise the study. SW devised the study, interpreted the results, and edited the manuscript. All authors contributed to the article and approved the submitted version.

## Conflict of Interest

The authors declare that the research was conducted in the absence of any commercial or financial relationships that could be construed as a potential conflict of interest.

## Publisher’s Note

All claims expressed in this article are solely those of the authors and do not necessarily represent those of their affiliated organizations, or those of the publisher, the editors and the reviewers. Any product that may be evaluated in this article, or claim that may be made by its manufacturer, is not guaranteed or endorsed by the publisher.

## Footnotes

1
https://www.diversityarrays.com/

2
https://phytozome.jgi.doe.gov/pz/portal.html#!info?alias=Org_Sbicolor

3
https://plants.ensembl.org/Oryza_sativa/Info/Index
