# Supplementary material for: Genetic Architecture of Novel Sources for Reproductive Cold Tolerance in Sorghum
Source: Front Plant Sci. 2021 Nov 24;12:772177. doi: 10.3389/fpls.2021.772177 (PMC8652046; doi:10.3389/fpls.2021.772177)
Supplement: Supplementary file 2 [file Presentation_1.pptx]

## Slide 1
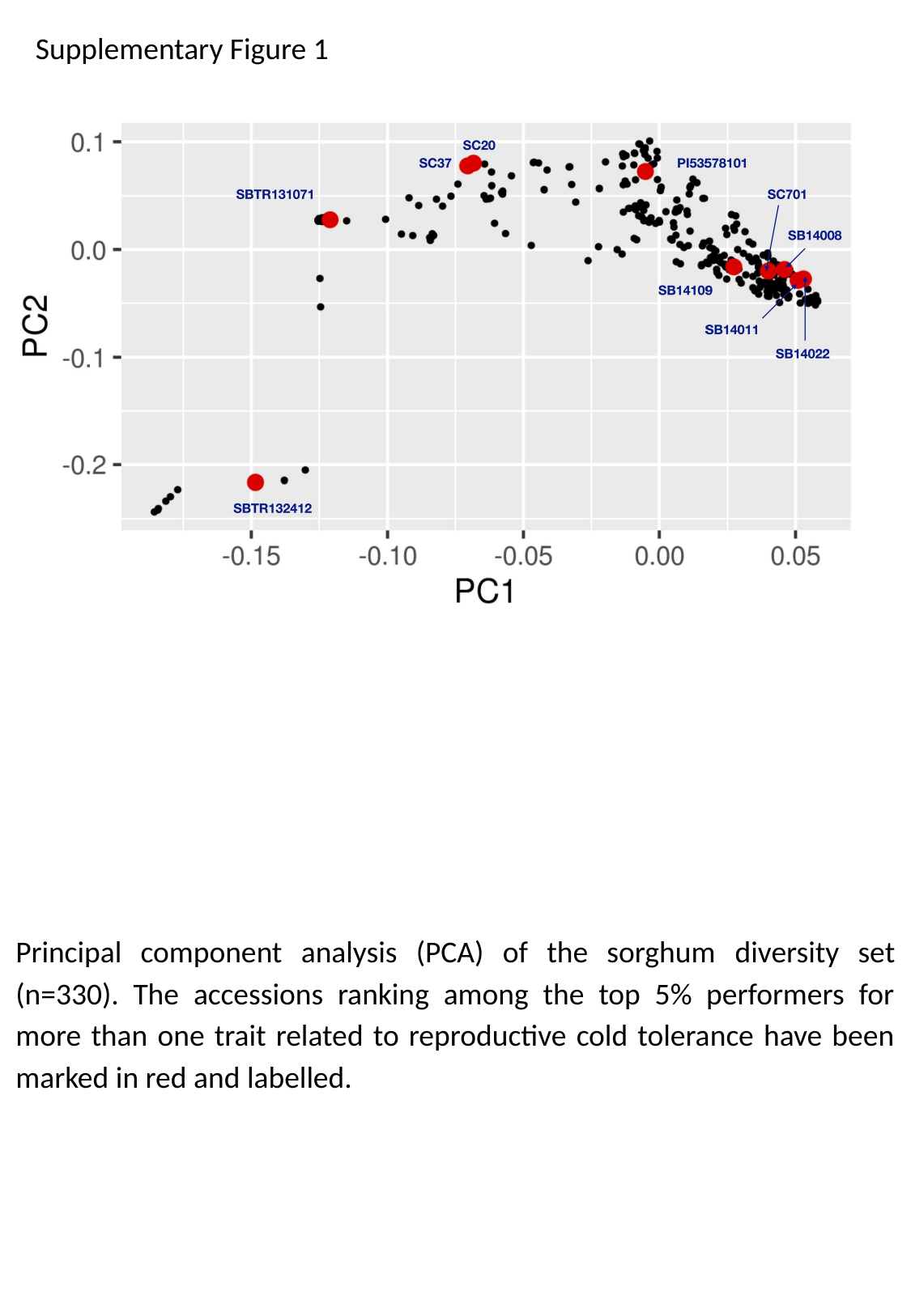

Supplementary Figure 1
Principal component analysis (PCA) of the sorghum diversity set (n=330). The accessions ranking among the top 5% performers for more than one trait related to reproductive cold tolerance have been marked in red and labelled.

## Slide 2
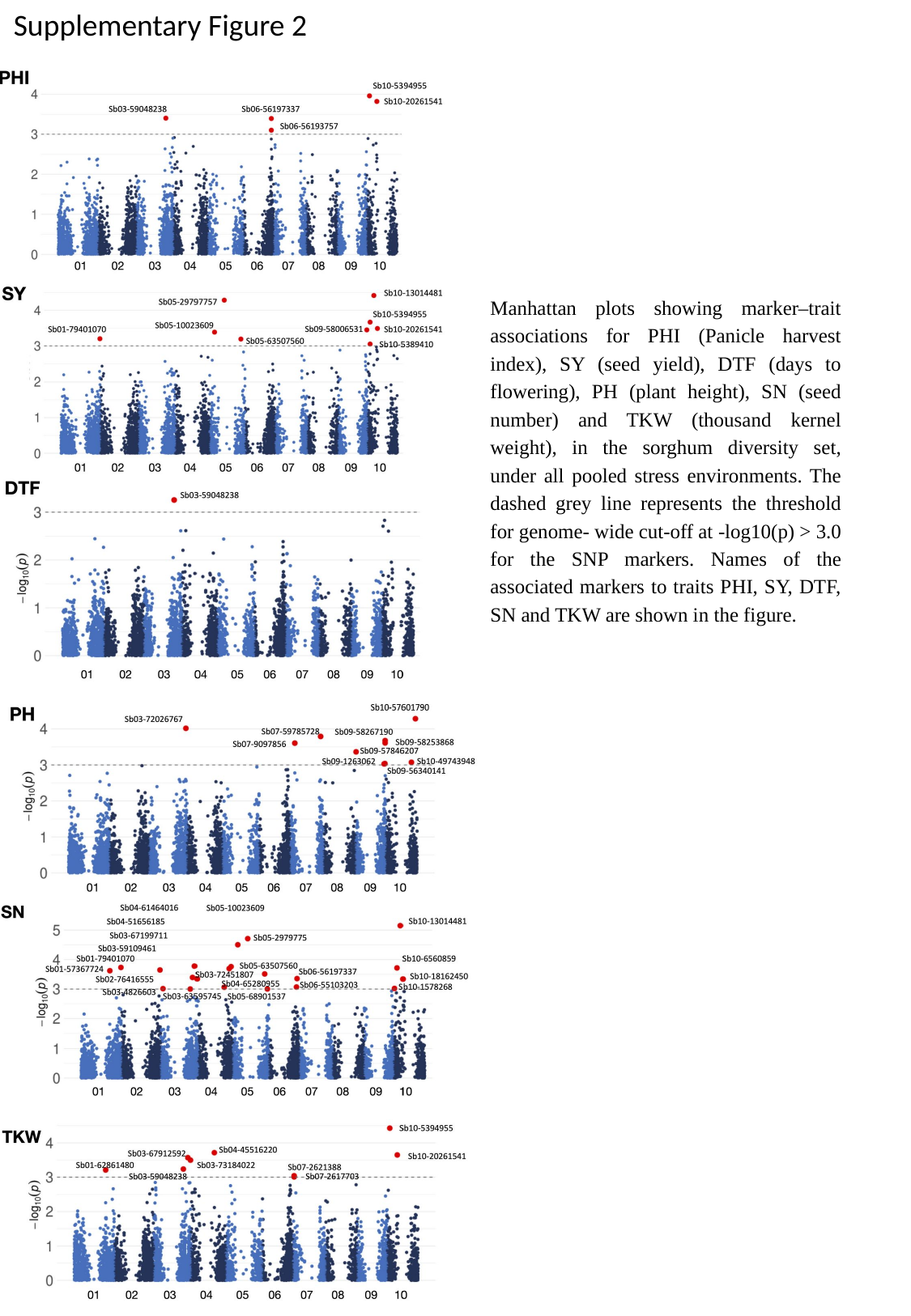

Supplementary Figure 2
Manhattan plots showing marker–trait associations for PHI (Panicle harvest index), SY (seed yield), DTF (days to flowering), PH (plant height), SN (seed number) and TKW (thousand kernel weight), in the sorghum diversity set, under all pooled stress environments. The dashed grey line represents the threshold for genome- wide cut-off at -log10(p) > 3.0 for the SNP markers. Names of the associated markers to traits PHI, SY, DTF, SN and TKW are shown in the figure.

## Slide 3
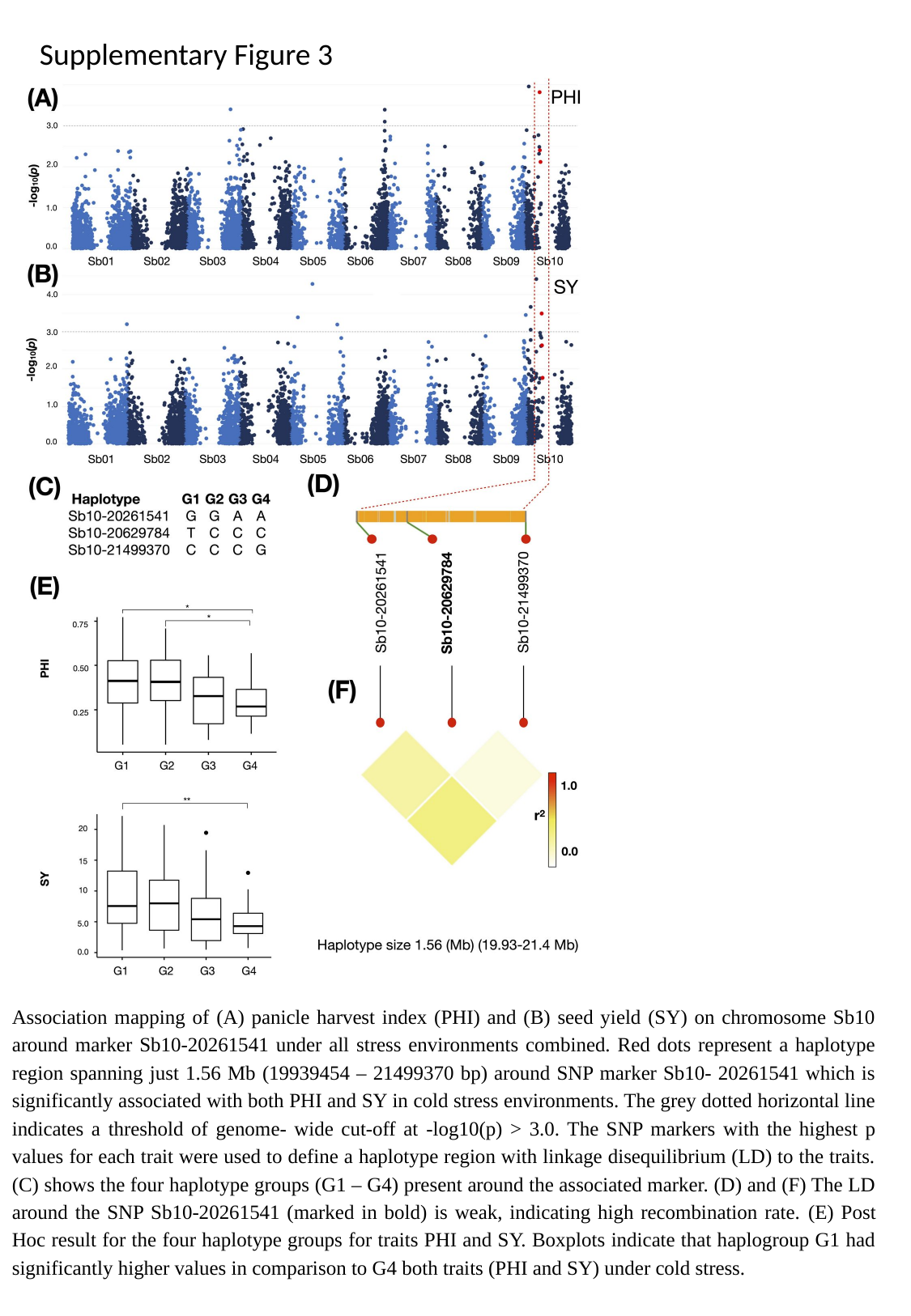

Supplementary Figure 3
Association mapping of (A) panicle harvest index (PHI) and (B) seed yield (SY) on chromosome Sb10 around marker Sb10-20261541 under all stress environments combined. Red dots represent a haplotype region spanning just 1.56 Mb (19939454 – 21499370 bp) around SNP marker Sb10- 20261541 which is significantly associated with both PHI and SY in cold stress environments. The grey dotted horizontal line indicates a threshold of genome- wide cut-off at -log10(p) > 3.0. The SNP markers with the highest p values for each trait were used to define a haplotype region with linkage disequilibrium (LD) to the traits. (C) shows the four haplotype groups (G1 – G4) present around the associated marker. (D) and (F) The LD around the SNP Sb10-20261541 (marked in bold) is weak, indicating high recombination rate. (E) Post Hoc result for the four haplotype groups for traits PHI and SY. Boxplots indicate that haplogroup G1 had significantly higher values in comparison to G4 both traits (PHI and SY) under cold stress.

## Slide 4
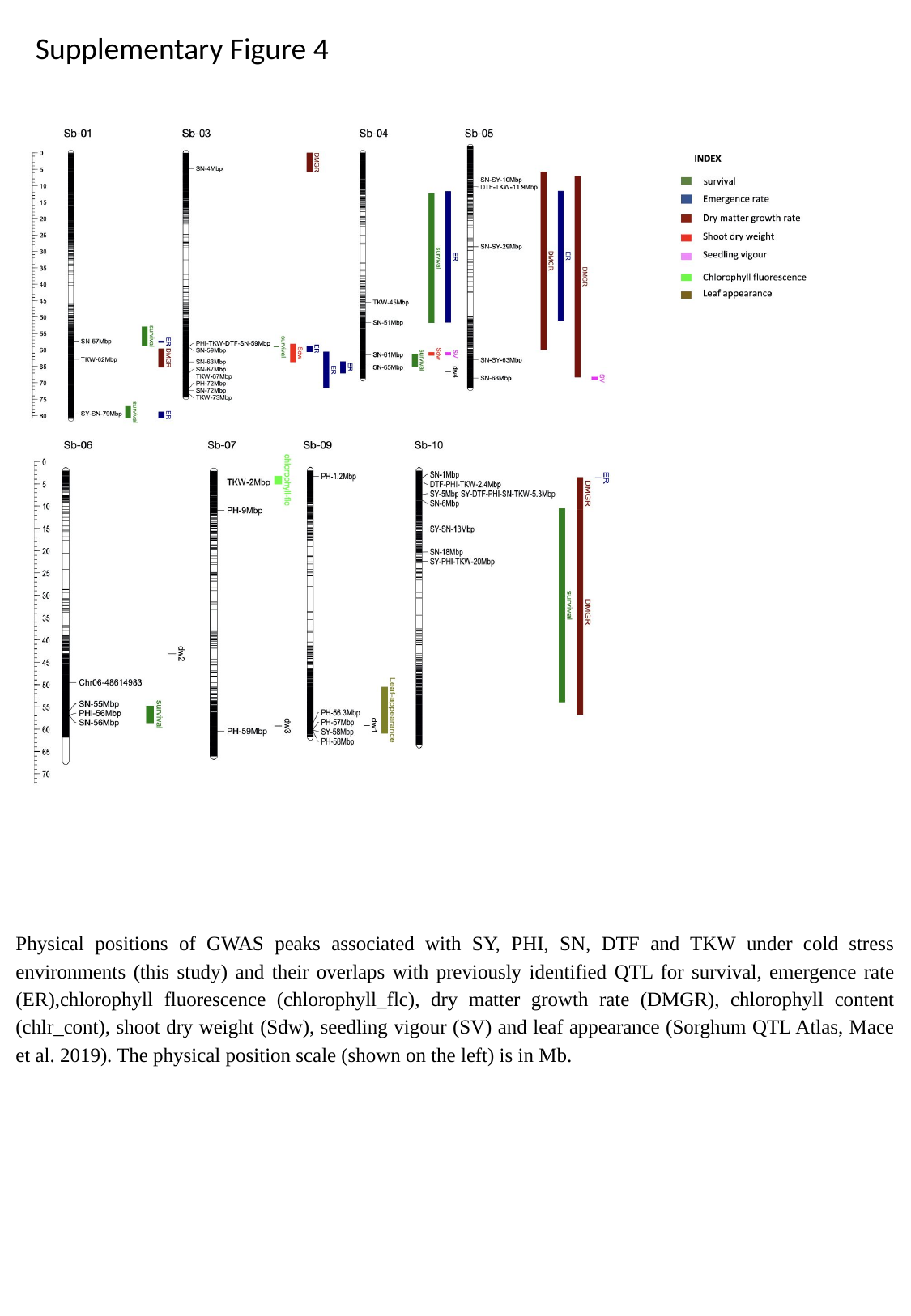

Supplementary Figure 4
Physical positions of GWAS peaks associated with SY, PHI, SN, DTF and TKW under cold stress environments (this study) and their overlaps with previously identified QTL for survival, emergence rate (ER),chlorophyll fluorescence (chlorophyll_flc), dry matter growth rate (DMGR), chlorophyll content (chlr_cont), shoot dry weight (Sdw), seedling vigour (SV) and leaf appearance (Sorghum QTL Atlas, Mace et al. 2019). The physical position scale (shown on the left) is in Mb.
